# Treadmill Intervention Attenuates the Cafeteria Diet-Induced Impairment of Stress-Coping Strategies in Young Adult Female Rats

**DOI:** 10.1371/journal.pone.0153687

**Published:** 2016-04-21

**Authors:** Igor Cigarroa, Jaume F. Lalanza, Antoni Caimari, Josep M. del Bas, Lluís Capdevila, Lluís Arola, Rosa M. Escorihuela

**Affiliations:** 1 Institut de Neurociències, Departament de Psiquiatria i Medicina Legal, Universitat Autònoma de Barcelona, Barcelona, Spain; 2 Carrera de Kinesiología, Facultad de Salud, Universidad Santo Tomás, Los Ángeles, región del Bio-Bio, Chile; 3 Grup de Recerca en Nutrició i Salut (GRNS). Centre Tecnològic de Nutrició i Salut (CTNS), TECNIO, CEICS, Reus, Spain; 4 Laboratori de Psicologia de l’Esport, Departament de Psicologia Bàsica, Universitat Autònoma de Barcelona, Barcelona, Spain; 5 Departament de Bioquímica i Biotecnologia, Nutrigenomics Research Group, Universitat Rovira i Virgili, Tarragona, Spain; 6 Centre Tecnològic de Nutrició i Salut (CTNS), TECNIO, CEICS, Reus, Spain; Universidad Pablo de Olavide, Centro Andaluz de Biología del Desarrollo-CSIC, SPAIN

## Abstract

The current prevalence of diet-induced overweight and obesity in adolescents and adults is continuously growing. Although the detrimental biochemical and metabolic consequences of obesity are widely studied, its impact on stress-coping behavior and its interaction with specific exercise doses (in terms of intensity, duration and frequency) need further investigation. To this aim, we fed adolescent rats either an obesogenic diet (cafeteria diet, CAF) or standard chow (ST). Each group was subdivided into four subgroups according to the type of treadmill intervention as follows: a sedentary group receiving no manipulation; a control group exposed to a stationary treadmill; a low-intensity treadmill group trained at 12 m/min; and a higher intensity treadmill group trained at 17 m/min. Both the diet and treadmill interventions started at weaning and lasted for 8 weeks. Subjects were tested for anxiety-like behavior in the open field test and for coping strategies in the two-way active avoidance paradigm at week 7 and were sacrificed at week 8 for biometric and metabolic characterization. CAF feeding increased the weight gain, relative retroperitoneal white adipose tissue (RWAT %), and plasma levels of glucose, insulin, triglycerides and leptin and decreased the insulin sensitivity. Treadmill intervention partially reversed the RWAT% and triglyceride alterations; at higher intensity, it decreased the leptin levels of CAF-fed animals. CAF feeding decreased the motor activity and impaired the performance in a two-way active avoidance assessment. Treadmill intervention reduced defecation in the shuttle box, suggesting diminished anxiety. CAF feeding combined with treadmill training at 17 m/min increased the time spent in the center of the open field and more importantly, partially reversed the two-way active avoidance deficit. In conclusion, this study demonstrates that at doses that decreased anxiety-like behavior, treadmill exercise partially improved the coping strategy in terms of active avoidance behavior in the CAF-fed animals. This effect was not observed at lower doses of treadmill training.

## Introduction

The current prevalence of overweight and obesity has reached epidemic levels in adolescents [1], and it is over 30% in adults [2]. In addition to affecting metabolic and cardiovascular disorders, obesity is increasingly associated with psychological and psychiatric disorders [3–5]. Obesity has been connected to anxiety, depression and bipolar disorders [4,6]. Moreover, anxiety, stress and emotional instability have been demonstrated to alter food consumption and eating behavior, thus contributing to overweight and obesity at all ages [5].

Among the different animal models developed to investigate obesity disorders [7,8], the palatable cafeteria (CAF) diet is a diet-induced obesity (DIO) model in which rodents are offered the highly palatable and energy dense foods regularly consumed by humans with concurrent free access to standard chow (ST) and water [9]. The CAF diet promotes voluntary hyperphagia [10] and weight gain, increases fat mass, alters the circulating levels of glucose and insulin [11,12], and induces liver and adipose tissue inflammation [9]. Regarding environmental and psychological factors, this obesogenic diet increases the hedonic properties of the ‘wanted’ food and the motivation to consume this food [13] and alters eating behavior [14], thus providing a robust rodent model of the metabolic and behavioral processes underlying diet-induced human obesity [9,15] and metabolic syndrome [16].

Palatable food consumption has decreased anxiety and the stress response in rodents [17,18], and different studies have reported diminished anxiety-like behavior in rats that preferred a palatable high-fat diet [19], in the offspring of CAF-fed dams [20], in CAF-fed rats previously subjected to maternal separation [21], and in late adolescent and adult rats fed a palatable diet [12,22]. However, all the above studies evaluated the anxiety-like behavior by using unconditioned and/or ethological models. Among the two main categories for measuring behavioral anxiety in rodents (i.e., unconditioned *vs* conditioned response tests), the unconditioned tests evaluate coping strategies under novel threatening conditions (i.e., environments) that approximate to the natural open or unprotected spaces shown to elicit anxiety [23,24]. In those conditions, the inappropriate levels of risk assessment activity induced by a threatening environment would be analogous to some human anxiety symptoms of phobic tendencies, hypervigilance, scanning and excessive worries [25]. In contrast, the conditioned response tests evaluate coping strategies by pairing a neutral stimulus with a threatening stimulus (electric shock) to promote specific avoidance/escape or defensive behavioral responses (i.e., active or passive) to escape or avoid the shock [26]. The present study goes further and evaluates the performance of DIO rats in a conditioned response test, the two-way (shuttle-box) active avoidance test, which is based on a conflict situation that involves two opposing (passive *vs* active) responses.

Exercise prevented overweight in rats fed standard [27] and high-fat diets [28]. Exercise also normalized insulin sensitivity in obese rats [29], improved vascular function and ameliorated high-fat diet-induced metabolic dysfunction, including abdominal fat content, systemic inflammatory cytokine levels, glucose tolerance and insulin resistance [30–32]; these effects were in addition to the increase in the mRNA expression and protein levels markers related to synaptic plasticity and cognitive function [33]. More recent evidence based on rodents and humans studies supports a bi-directional relationship between obesity and anxiety, depressive or bipolar disorders; thus, effective weight-loss interventions might contribute to improved psychological health [4]. For example, in rodents, treadmill exercise reduced anxiety-like behavior in the elevated plus-maze and open field unconditioned tests [34,35], improved the coping strategies in the two-way active avoidance conditioned paradigm [36] and reduced the hormonal response to acute stress [37]. Overall, exercise practice seems to decrease anxiety-like behavior when administered under certain protocol conditions.

On the basis of the above studies, the aims of the present study were to evaluate the following: 1) the effects of CAF diet-induced obesity on the two-way active avoidance conditioned response and 2) whether two different treadmill running procedures (i.e., low and higher intensity) combined with the CAF diet could modify the behavior of young adult female rats in the open field test and the two-way (shuttle-box) active avoidance performance. Furthermore, the effects of treadmill running on food consumption, nutrient and energy intake, and biometric and metabolic plasma parameters, including leptin levels, were also evaluated in ST- and CAF-fed rats.

The main advantages of our model over other models of exercise are the following: a) with a treadmill model, the experimenter can adjust the duration and intensity of exercise and hence control for the heterogeneity reported in other models (e.g., voluntary exercise training [38]) and b) with two control groups, a sedentary group (SED) receiving no manipulation and a control group (CON) exposed to a stationary treadmill under the same conditions as for the treadmill (TM) group, the influence of other variables, such as daily handling and mere exposure to the treadmill apparatus, can be detected [37,39]. To our knowledge, no previous studies have examined the intersection between exercise and palatable CAF feeding on conditioned anxiety test results in a well-controlled animal model.

## Materials and Methods

### Ethics statement

The experimental protocol was approved by the *Generalitat de Catalunya (DAAM 6836)*, following the ‘Principles of laboratory animal care’, and was carried out in accordance to the European Communities Council Directive (86/609/EEC).

### Animals and housing conditions

The animals were female Sprague-Dawley rats bred and raised at the main animal facility of the Universitat Autònoma de Barcelona. They were weaned at 21–23 days of age, housed with 2 animals per cage (same experimental group) in standard macrolon cages (40 cm in length x 23 cm in width x 18 cm in depth.), fed with *ad libitum* access to the corresponding diet (see below) and maintained in conditions of standard temperature (21 ± 1°C) and humidity (50±10%) with a 12 h-12 h light-dark cycle (lights on at 08:00 h). Animals were randomly assigned to eight groups with comparable total body weights before the beginning of the treadmill intervention: sedentary-standard diet (SED-ST), sedentary-cafeteria diet (SED-CAF), control-standard diet (CON-ST), control-cafeteria diet (CON-CAF), treadmill-low intensity-standard diet (TML-ST), treadmill-low intensity-cafeteria diet (TML-CAF), treadmill-higher intensity-standard diet (TMH-ST), and treadmill-higher intensity-cafeteria diet (TMH-CAF).

### Cafeteria diet

Beginning at weaning (at day 21 of life) and for a duration of 8 weeks, the rats were fed either standard chow (ST; Harland 5,2) or a cafeteria diet (CAF) with the following components (quantity per rat): bacon or frankfurter (8–12 g), biscuit with pâté (12–15 g), biscuit with cheese (10–12 g), muffins or ensaïmada (pastry) (8–10 g), carrots (6–8 g), milk with sugar (220 g/l; 50 ml), water (*ad libitum*), and ST chow. The ST chow had a calorie breakdown of 24% protein, 18% fat and 58% carbohydrates, whereas the calorie breakdown of the CAF diet was: 10% proteins; 41% fat; and 49% carbohydrates. The animals were fed *ad libitum*, the food was renewed daily [12], and the amounts of each component that were eaten were determined twice weekly. The amounts consumed were the differences between the weight of the fluids and foods allocated to a cage and those remaining 24 hr after. We assumed that the two animals of the same cage consumed half of the difference [40].

### Treadmill intervention

We included two exercise groups of low and higher intensities (12 m/min and 17 m/min, respectively). The treadmill consisted of three parallel runways (45 cm x 11 cm x 12 cm, Columbus Instruments, USA) without inclination. The procedure was similar to the one described in Lalanza et al. [37]. Briefly, training sessions started after weaning and continued for 8 weeks. They were conducted 4–5 days per week in the colony room and lasted 30 min. On the first day, the subjects were habituated to the treadmill for 30 min (0 m/min). On the next day, training began gently, and the intensity of the treadmill speed was gradually increased until it reached a maximum intensity of 12 m/min (TML groups) or 17 m/min (TMH groups); these speeds were maintained until the end of the experiment. Neither electrical shock nor physical prodding was used to motivate the animals. The control (CON) rats stayed on a stationary treadmill (0 m/min) for the same number of sessions and the same amount of time as the TML and TMH rats. The sedentary (SED) rats remained in their cages. All rats were weighed weekly.

### Behavioral experiments

Behavioral experiments were carried out during the 7^th^ week of diet administration, when the animals were 10 weeks of age. Treadmill training was interrupted during behavioral testing. All experiments were carried out in an isolated experimental room with dim lighting. Animals were tested for locomotion and activity in an open field (OF) and for two-way active avoidance learning in a shuttle box (SB). The apparatus was cleaned with a 20% ethanol solution after each rat.

#### Open field

The open field experiment was carried out in the morning (between 9:00 am and 16:00 pm) in a beige plywood arena (diameter 83 cm) surrounded by a white plywood wall (height 34 cm). The rats were individually housed in a cage with clean sawdust for 30 minutes before being placed in the open field. Then, the animals were individually placed in the center of the arena and allowed to explore the apparatus for 30 min. The total distance travelled and the time spent in the central area were recorded using a HD camera (JVC) and then measured and analyzed using video tracking software (ANY-Maze, San Diego Instruments).

#### Shuttle box (SB)

The shuttle-box apparatus (Panlab, S.L.) was divided into two equally sized compartments (25 cm x 25 cm x 25 cm) connected by an opening door (8 cm wide and 10 cm high). Animals were placed in the shuttle box for a habituation period of 10 min before the start of the 40-trial session. Each trial consisted of 10 s of simultaneously presentation of a light (7 W, 10 s) and a tone (2400 Hz at 40 dB) as a conditioned stimulus (CS); the stimulus was immediately followed by a scrambled electric shock (0.6 mA, 20 s), which was administered through the metal grid floor of the box (unconditioned stimulus, US). The animal had to learn to change between the two compartments of the shuttle box (separated by an open door) to escape or avoid the shock. The CS or US was terminated when the animal crossed to the other compartment; crossings that occurred during the CS, and thus before the US onset, were considered an avoidance response. Once a crossing had been made and/or the shock discontinued, an inter-trial interval (ITI; 40 s) was presented. Escape latencies, the number of avoidance responses, defecations and the number of crossings made during the habituation period and during inter-trial intervals were scored.

### Physiological and metabolic variables

Body weight was monitored weekly over 8 weeks. Food was withdrawn 12–14 h before sacrifice. Animals were sacrificed by beheading, and the total blood was collected. Serum was obtained by centrifugation at 4°C and 2000 g for 15 minutes and stored at -80°C until further use. The retroperitoneal white adipose tissue (RWAT) and liver were dissected and weighed. The relative liver weight and relative RWAT weight were calculated following the formula (100* tissue weight/body weight) and were thus expressed as a percentage of the total body weight.

#### Plasma analysis

Enzymatic colorimetric kits were used for the determination of serum glucose, triglyceride, cholesterol (QCA, Barcelona, Spain) and non-esterified free fatty acid (NEFA) (WAKO, Neuss, Germany) levels. The serum insulin and leptin levels were measured using a mouse/rat insulin ELISA kit and a rat leptin ELISA kit, respectively, (Millipore, Barcelona, Spain) following the manufacturer’s instructions.

#### R-QUICKI analysis

The insulin sensitivity was assessed by the revised quantitative insulin sensitivity check index (R-QUICKI) using the following formula: 1/[log insulin (μU/mL) + log glucose (mg/dL) + log FFA (mmol/l)] [41,42].

### Statistical analysis

The data were analyzed using the “Statistical Package for Social Sciences” (IBM SPSS Statistics, v 21). Two-way ANOVA analysis (2x2 factorial designs: diet × treadmill intervention) was used to evaluate the average daily fluid (log transformed to homogenate variances), solid and nutrient consumptions, biometric and plasma parameters and was also used for, the open field data and the number of crossings and defecations in the shuttle box. When one or both main effects were statistically significant, one-way ANOVA followed by the least significance difference (LSD) test was used to determine treatment differences between groups. Independent Student’s t-test for comparisons across groups was applied when necessary. The number of avoidances and the average escape latencies over shuttle-box avoidance acquisition were analyzed by repeated measures ANOVA with trials (4 blocks of 10-trials each) as within-subject factors, and diet and treadmill intervention were used as between-subject factors. A paired t-test was used for comparisons between the first and fourth 10-trial blocs of the shuttle-box variables. Linear relationships between key variables were tested using Pearson’s correlation coefficients. The cumulative sugary milk intake and carbohydrate consumption in CAF-fed animals were analyzed by a repeated measures ANOVA with weeks (8) as a within-subject factor and treadmill intervention as a between-subject factor. All values are expressed as the mean ± standard error for the mean (SEM). Statistical significance was set at p<0.05 for all tests.

## Results

### Change in the average daily consumption of liquids and food in CAF- and ST-fed rats

CAF-fed animals consumed less water and chow than the ST-fed groups (diet: F(1,82) = 236.15, p<0.001 and F(1,82) = 2273.1, p<0.001, respectively; Table 1) but more total fluid (log transformed) and total solid food (F(1,82) = 1091.74, p<0.001 and F(1,82) = 24.02, p<0.001, respectively; Table 1). In regard to the nutrients, the CAF-fed rats consumed quadruple the amount of fat (F(1,82) = 1801.3, p<0.001; Table 1) and carbohydrates (F(1,82) = 977.2, p<0.001; Table 1) but less protein (F(1,82) = 78.82, p<0.001; Table 1) and fiber (F(1,82) = 148.213, p<0.001; Table 1) compared with the ST-fed animals. The different eating behavior between ST- and CAF-fed rats was translated into considerable changes in energy intake, with the CAF-fed animals consuming triple the amount of Kcal compared with that of the ST-fed animals (F(1,82) = 971.1, p<0.001; Table 1).

**Table 1:** Average daily intake (mean ± SEM) of standard chow, fluids, nutrients and energy over the 8 weeks of the experiment.

	SED_ST	CON_ST	TML_ST	TMH_ST	SED_CAF	CON_CAF	TML_CAF	TMH_CAF	two-way ANOVA
**Chow (g/d)**	17.11±0.41^A^	17.49±0.28^A^	18.66±0.71^B^	16.43±0.21^A^	3.88±0.47^C^	4.68±0.23^C^a	4.48±0.14^C^a	3.73±0.14^C^a	*D*,*T*
**Total solid food (g/d)**	17.11±0.41^AB^	17.49±0.28^AB^	18.66±0.71^BC^	16.43±0.21^A^	17.83±0.58^AB^	18.58±1.12^BC^	20.42±0.79^CD^	22.05±0.63^D^ab	*D*,*TDxT*
**Water (g/d)**	25.66±1.10^A^	27.38±0.46^A^	27.83±1.00^A^	26.19±1.09^A^	12.17±1.55^B^	14.02±1.53^B^a	13.33±1.86^B^a	11.91±1.16^B^a	*D*
**Milk (g/d)**	---	---	---	---	62.29±2.59^A^	61.64±2.26^AB^	54.27±2.40^BC^	51.69±3.72^C^	
**Total fluid (g/d)**	25.66±1.10^A^	27.38±0.46^A^	27.83±1.00^A^	26.19±1.09^A^	74.46±2.99^B^	75.66±2.51^B^a	66.95±2.67^BC^a	64.15±4.03^C^ab	*D*
**KCAL**	58.16±1.38^A^	59.45±0.95^A^	63.43±2.40^A^	55.85±0.72^A^	190.3±7.8^B^	185.0±5.4^BC^a	176.5±8.2^BC^a	168.9±8.6^C^ab	*D*
**Protein**	3.22±0.08^A^	3.29±0.05^AB^	3.51±0.13^B^	3.09±0.04^A^	2.74±0.14^C^	2.81±0.04^C^a	2.75±0.06^C^a	2.64±0.08^C^a	*D*,*T*
**Fat**	1.03±0.02^A^	1.05±0.02^A^	1.12±0.04^A^	0.99±0.01^A^	3.92±0.09^B^	4.11±0.11^B^a	3.96±0.16^B^a	3.94±0.17^B^a	*D*
**Carbohydrate**	8.55±0.20^A^	8.75±0.14^A^	9.33±0.35^A^	8.21±0.11^A^	37.68±1.64^B^	36.2 ±1.23^B^a	34.37±1.78^BC^a	32.47±1.88^C^ab	*D*
**Fibre**	0.65±0.02^A^	0.66±0.01^AB^	0.71±0.03^B^	0.62±0.01^A^	0.45±0.02^C^	0.48±0.03^CD^a	0.52±0.02^DE^a	0.54±0.01^E^ab	*D*,*T*,*DxT*

Rats were fed from weaning (at day 21 of life) for 8 weeks with a ST or a CAF (g/d = grams per day) diet and trained on a treadmill at different intensities (CON: 0 m/min; TML: 12 m/min or TMH: 17 m/min) during the same period. SED rats were left undisturbed in the home cage except for cage cleaning and body weight measurements. Data show average daily intake expressed as gram/day/rat. The statistical comparison was performed by two-way ANOVA (2 diet (ST, CAF) x 4 treatment (SED, CON, TML, and TMH)). *D*: the effect of the type of diet, T: the effect of the treatment, DxT: the effect of the diet x treatment interaction (two-way ANOVA, p<0.05).^ABC^ Mean values within a row with dissimilar capital letters were significantly different among groups (one-way ANOVA and LSD post hoc comparison, p<0.05). a p<0.05 vs the corresponding ST group (same treatment); b p<0.05 vs the corresponding SED group (same diet) (p<0.05 Mann-Whitney test).

Treadmill intervention increased the chow and total solid food (F(3,82) = 5.94, p = 0.001 and F(3,82) = 5.38, p<0.01, respectively; Table 1), with the latter effect being greater in CAF-fed animals (diet x treadmill interaction: F(3,82) = 3.904, p<0.05; Table 1). Treadmill intervention also decreased the protein consumption (F(3,82) = 4.597, p<0.05; Table 1) but increased the fiber consumption (F(3,82) = 4.89, p<0.01; Table 1); this latter effect was greater in the CAF-fed animals (diet x treadmill interaction: F(3,82) = 2.94, p<0.05; Table 1). Treadmill intervention residually decreased the total fluid consumption in the CAF-fed animals (diet x treadmill interaction: F(3,82) = 2.652, p = 0.055; Table 1). This effect was mainly caused by a significant decrease in milk intake in both the TML-CAF and TMH-CAF groups compared with the SED-CAF and/or CON-CAF animals (one-way ANOVA, p<0.05; Table 1). The TMH-CAF group had the greatest increase in total solid food consumption, reaching significant differences compared with all the other groups except TML-CAF (p<0.05; Table 1). However, the TMH-CAF animals showed decreased carbohydrate and kcal consumption compared with that of the SED-CAF rats and increased fiber consumption when compared with that of the SED-CAF and CON-CAF groups (p<0.05, Table 1).

### Impact of CAF diet and treadmill intervention on the body and tissue weights

At the end of the study, the four groups of CAF-fed animals had become overweight compared with the ST-fed controls (Table 2; F(1,81) = 98.98, p<0.001), which could be attributed to their higher daily energy intake (Table 1). Importantly, the increase in RWAT weight (expressed as percentage of body weight) found in the CAF-fed animals was partially counteracted by treadmill intervention at low and higher intensity (significant diet x treadmill interaction: F(3,81) = 3.022, p<0.05), Table 2); the observed effect was similar in the two groups of runners (20% decrease compared with the CON-CAF group; Table 2). No other diet and/or treadmill intervention effects appeared in the relative RWAT or liver weight.

**Table 2:** Biometric and plasma parameters (mean ± SEM) of sedentary (SED), control (CON) and treadmill-trained (TML: 12 m/min; TMH: 17 m/min) female rats fed a standard chow diet (ST) or a cafeteria (CAF) + standard chow diet.

	SED-ST	CON-ST	TML-ST	TMH-ST	SED-CAF	CON-CAF	TML-CAF	TMH-CAF	two-way ANOVA
**Biometric parameters**									
Initial body weight (g)	63±2	61±2	62±2	61±1	60±1	63±1	61±2	61±2	
Final body weight (g)	266±6^a^	252±6^a^	275±11^a^	253±6^a^	320±13^b^	327±6^b^	323±9^b^	309±9^b^	*D*
Body weight gain (g)	204±5^a^	191±7^a^	212±10^a^	192±6^a^	260±12^b^	264±5^b^	262±9^b^	248±9^b^	*D*
Liver weight (%)	2.61±0.04	2.59±0.04	2.72±0.04	2.64±0.07	2.50±0.06	2.56±0.06	2.63±0.05	2.60±0.11	
RWAT weight (%)	1.68±0.17^ab^	2.08±0.10^ab^	2.13±0.16^a^	1.61±0.17^b^	4.15±0.28^cd^	4.59±0.15^c^	3.69±0.19^d^	3.81±0.23^d^	*D*, *T*, *DxT*
**Plasma parameters**									
Glucose (mmol/L)	6.50±0.17^ab^	6.13±0.34^a^	6.63±0.14^ab^	6.45±0.31^ab^	6.99±0.41^b^	7.16±0.31^b^	7.06±0.22^b^	7.21±0.15^b^	*D*
Insulin (mmol/L)	11.5±1.3^a^	10.3±0.7^a^	12.6±1.0^a^	9.4±0.6^a^	28.7±4.9^b^	31.8±5.5^b^	52.1±11.6^c^	40.8±9.2^bc^	*D*
R-QUICKI	0.33±0.01^a^	0.33±0.00^a^	0.33±0.00^a^	0.33±0.01^a^	0.29±0.01^b^	0.29±0.01^b^	0.28±0.01^b^	0.29±0.01^b^	*D*
Triglycerides (mmol/L)	0.88±0.09^a^	0.73±0.06^a^	0.75±0.05^a^	0.74±0.08^a^	1.68±0.22^bc^	1.81±0.20^b^	1.33±0.11^c^	1.0±0.14^a^	*D*, *T*, *DxT*
NEFAs (mmol/L)	0.89±0.05	0.92±0.06	0.83±0.04	0.93±0.06	0.93±0.07	0.93±0.05	0.79±0.06	0.74±0.05	
Leptin (ng/mL)	3.09±0.36^a^	2.94±0.23^a^	3.48±0.51^a^	2.94±0.49^a^	16.2±2.7^bc^	18.5±2.7^b^	15.4±1.7^bc^	12.1±2.6^c^	*D*

Female rats were randomly assigned to eight groups depending on the diet and the treadmill training during the 8 week period: sedentary-standard diet (SED-ST), sedentary-cafeteria diet (SED-CAF), control-standard diet (CON-ST), control-cafeteria diet (CON-CAF), treadmill-low intensity-standard diet (TML-ST), treadmill-low intensity-cafeteria diet (TML-CAF), treadmill-higher intensity-standard diet (TMH-ST), and treadmill-higher intensity-cafeteria diet (TMH-CAF). Relative liver weight and relative RWAT weight were calculated following the formula (100* tissue weight/body weight) and expressed as a percentage of the total body weight. The data are given as the mean ± SEM (n = 9–12). The statistical comparison among the different experimental groups was performed by two- and one-way ANOVA. *D*: the effect of the type of diet; *T*: the effect of the exercise (two-way ANOVA, p<0.05). ^abc^ Mean values within a row with dissimilar capital letters were significantly different among groups (one-way ANOVA and LSD post hoc comparison, p<0.05).

### Metabolic consequences of CAF feeding and treadmill intervention

CAF feeding for 8 weeks increased the circulating levels of glucose (χ^2^(7) = 13.90, p = 0.053) and insulin (χ^2^(7) = 50.13, p<0.001), and the increase was not counteracted by the treadmill intervention performed either at low or at higher intensity (Table 2). At the end of the study, the TMH-CAF group showed significantly lower serum levels of NEFAs than did the SED-CAF and the CON-CAF groups (p<0.05, Student’s t-test), and a similar trend was observed in the TML-CAF animals relative to the CON-CAF group (p = 0.079, Student’s t-test) (Table 2). Despite the drop in the circulating levels of NEFAs observed in the TML-CAF and TMH-CAF groups, treadmill intervention did not ameliorate the loss of insulin sensitivity (assessed by the R-QUICKI index) that resulted from the CAF diet. This fact could be explained, at least in part, by the higher insulin serum levels found in these two groups, especially in the TML-CAF animals; this group differed significantly from both the SED-CAF and the CON-CAF groups (Table 2). CAF-fed animals showed higher levels of serum triglycerides than did the ST-fed animals (diet: F(1,81) = 63.10, p<0.001), with this effect being gradually ameliorated by exercise (diet x treadmill interaction: F(3,81) = 2.664, p = 0.054) in the low-intensity runners (27% decrease in the TML-CAF group versus the CON-CAF animals) and almost counteracted in the TMH-CAF animals (45% decrease relative to the CON-CAF animals) (Table 2). In agreement with this last observation, correlation analyses considering all groups indicated negative correlations between treadmill intervention and the circulating levels of triglycerides (r = -0.284, p = 0.009). When the ST-fed and the CAF-fed groups were considered separately, this negative correlation was only observed in the CAF-fed groups, and the correlation was stronger than that obtained when considering all the groups (r = -0.451, p = 0.003). Increased circulating levels of leptin were found in the CAF-fed obese animals (diet: F(1,81) = 171.60, p<0.001). The treadmill intervention at higher intensity produced a significant decrease in circulating leptin (35% decrease in the TMH-CAF group versus the CON-CAF group, p<0.05; Table 2), which could be related to the lower adiposity observed in these animals. In the ST-fed groups, the treadmill intervention did not produce changes in any of the circulating parameters (Table 2).

### Correlations among RWAT weight, average carbohydrate consumption and serum leptin levels

A closer inspection of the correlational analysis when considering all the animals together revealed significant correlations between RWAT weight (%) and serum leptin levels (r = 0.846, p = 0.000), between RWAT weight (%) and the average daily intake of carbohydrates (r = 0.849, p = 0.000), and between serum leptin levels and the average daily intake of carbohydrates (r = 0.732, p = 0.000). When the ST-fed and the CAF-fed animals were considered separately, the RWAT weight (%) and serum leptin levels remained correlated for both ST-fed (r = 0.716, p = 0.000) and CAF-fed (r = 0.611, p = 0.000) animals. However, although the correlations between RWAT weight (%) and the average daily intake of carbohydrates (r = 0.504, p = 0.001) and those between circulating levels of leptin and the average daily intake of carbohydrates (r = 0.453, p = 0.005) were significant in the ST-fed animals, the correlation was not significant in the CAF-fed animals [RWAT weight (%) with carbohydrates (r = 0.156, p = 0.336); serum leptin levels with carbohydrates (r = 0.024, p = 0.883)].

### Impact of treadmill intervention on cumulative milk intake and carbohydrates consumption

**Fig 1:**
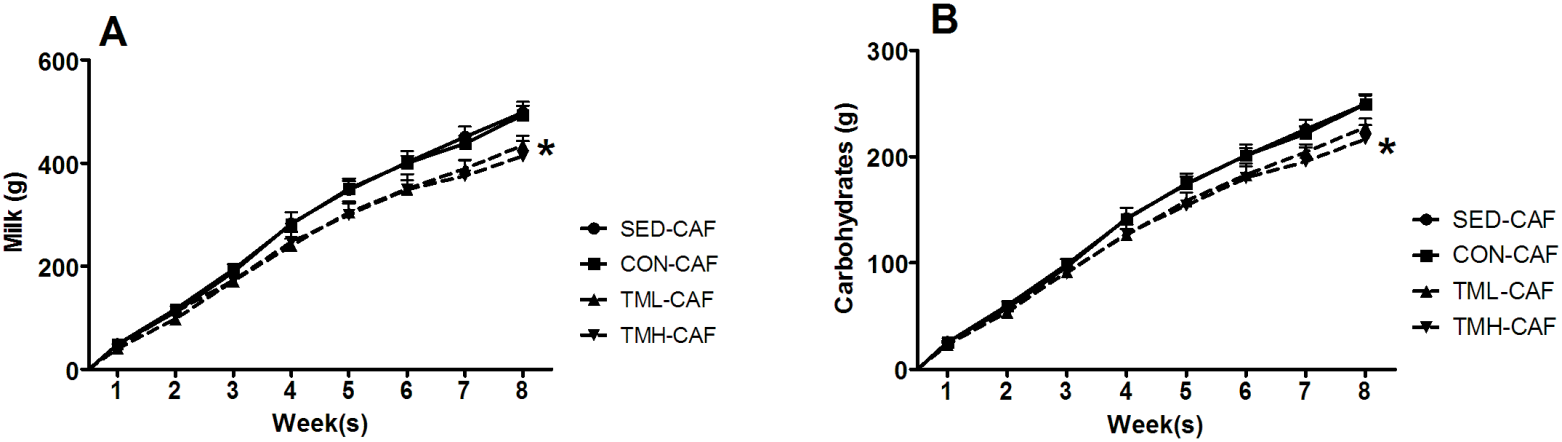
Cumulative daily A) milk and B) carbohydrate consumption over the 8 weeks of the experiment in CAF-fed groups. Rats were fed a CAF diet beginning at weaning (at day 21 of life) for 8 weeks and were trained on a treadmill at different intensities (CON: 0 m/min; TML: 12 m/min or TMH: 17 m/min) during the same period. SED rats were left undisturbed in the home cage except for cage cleaning and body weight measurements. The data represent the mean ± SEM (n = 10–12). CAF-fed animals had higher milk and carbohydrate consumption over time (p<0.001), but the cumulative intake of milk was lower in the TML-CAF and TMH-CAF groups, and the cumulative carbohydrate consumption was lower in the TMH-CAF group (*p<0.05 vs SED-CAF, after decomposition of the significant week x treadmill interaction).

The cumulative sugary milk intake and carbohydrate consumption over time in the CAF-fed animals are shown in Fig 1. The repeated measures analysis revealed a significant effect of week on both variables (milk: F(7,259) = 851.61, p<0.001; carbohydrates: F(7,266) = 3298.51, p<0.001), and a significant effect of week x treadmill intervention on carbohydrate: (F(21,266) = 2,905, p<0.05), thus indicating that the change in carbohydrate consumption over time varied depending on the treadmill intervention group. Decomposition of the significant interaction revealed that the TMH-CAF group had lower milk intake and carbohydrate consumption compared with the SED-CAF group at weeks 7 and 8. No other significant differences were found.

### Impact of CAF diet and treadmill intervention on the open field and two-way active avoidance performance

**Fig 2:**
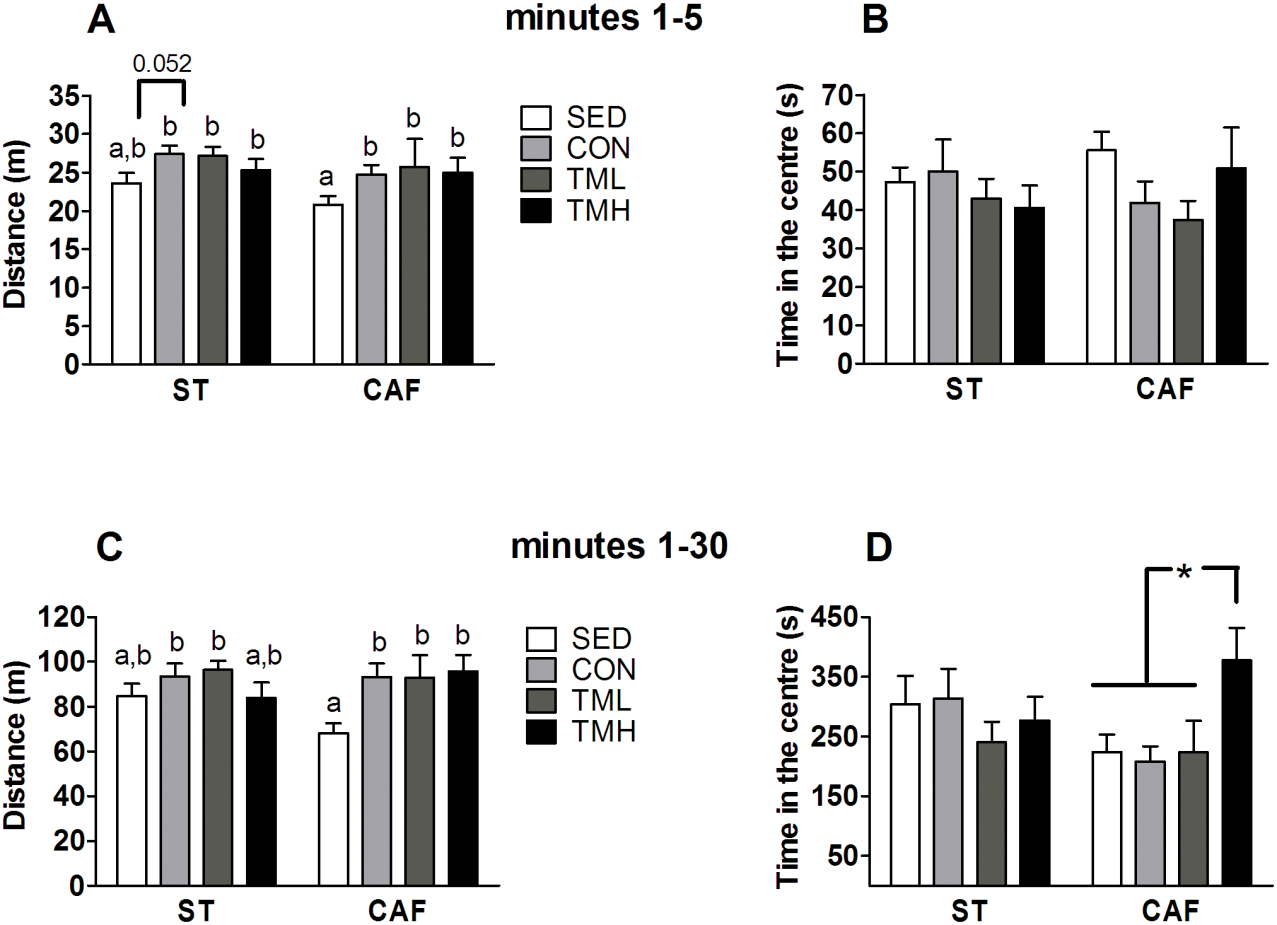
Distance travelled and time spent in the center of the open field during the first 5 min (A and B, respectively) and during the total 30 min (C and D, respectively). Rats were fed standard chow (ST) or a cafeteria diet (CAF) and/or trained on a treadmill (CON: 0 m/min; TML: 12 m/min; TMH: 17 m/min) beginning at weaning for a period of 8 weeks. The sedentary (SED) rats remained in their cages. The data represent the mean ± SEM (n = 10–12). CAF feeding reduced the distance travelled, whereas the treadmill intervention (handling and running) increased this value. The CAF diet and treadmill intervention at 17 m/min (TMH) strongly increased the time spent in the center. ^a, b^Mean values with different letters were significantly different between groups (two-way ANOVA and LSD post hoc comparison, p<0.05). p = 0.052 between the groups indicated in A; and * p<0.05 vs all CAF groups in D (independent Student’s t-test).

Overall, CAF feeding residually decreased the distance travelled during the first 5 minutes in the open field. The SED-CAF group traveled the shortest distance and differed from all the other groups, except the SED-ST group at 5 min (F(1,82) = 3.939, p = 0.054; Fig 2a) and the SED-ST and the TMH-ST groups at 30 min (Fig 2c). Overall, treadmill intervention increased the distance travelled, both during the initial 5 min (F(3,82) = 4.319, p<0.01; Fig 2a) and at the end of the 30-min (F(3,82) = 3.131, p<0.05; Fig 2c). The two-way ANOVA analysis did not show any overall significant effects of diet and/or treadmill intervention in the time spent in the center of the open field (Fig 2b and 2d), but a closer inspection revealed that TMH-CAF runners spent more time in the center during the 30 minutes of the open field test than did the other CAF-fed groups. This result indicates a decrease in the anxiety-like behavior in the open field for those animals (p<0.05 Student’s t-test; Fig 2d). No other significant effects were found in the open field analysis.

**Fig 3:**
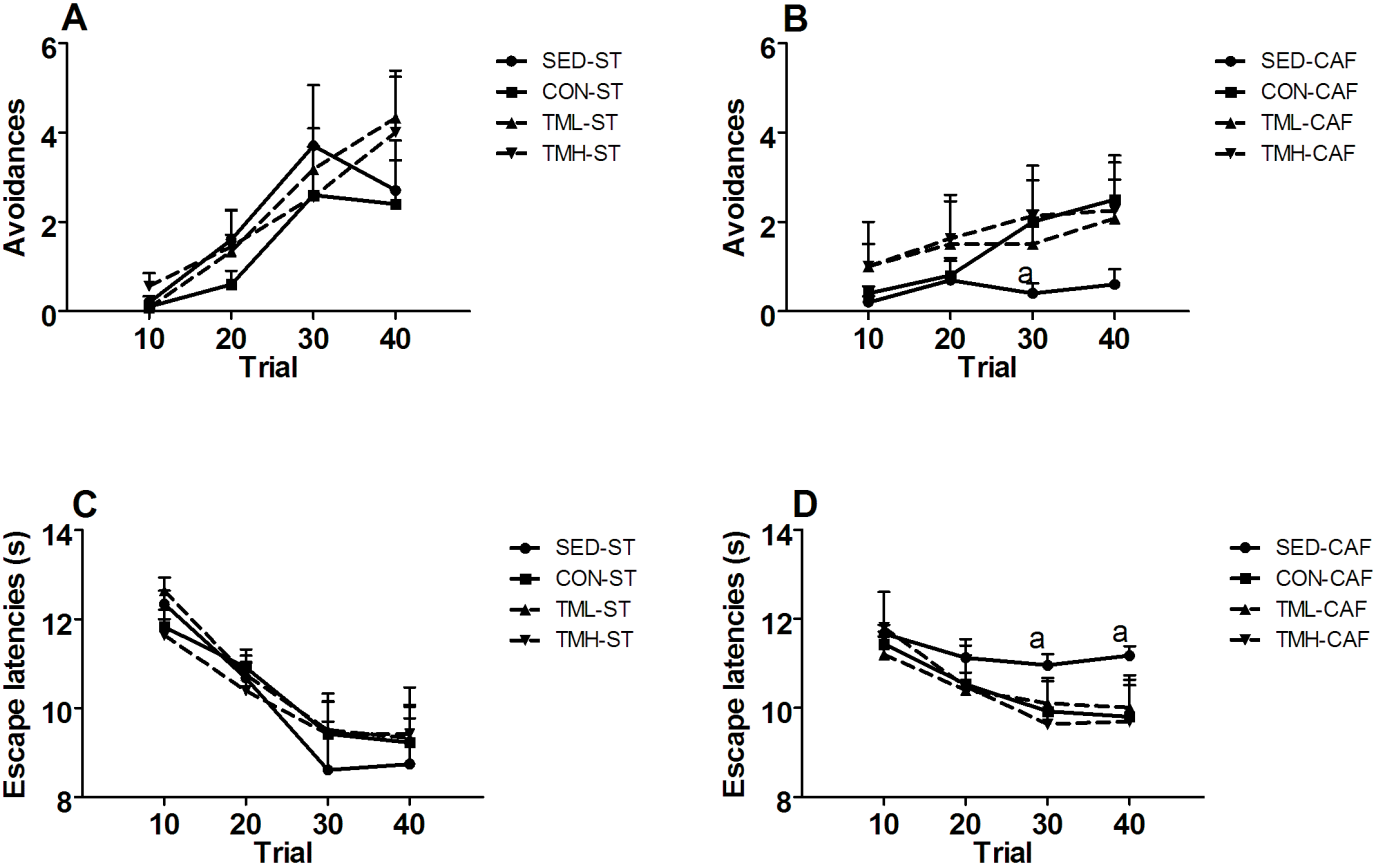
Avoidance responses (A-B) and average escape latencies (C-D) in blocks of ten trials of the two-way active avoidance session. Rats were fed standard chow (ST, left) or a cafeteria diet (CAF, right) and/or trained on a treadmill (CON: 0 m/min; TML: 12 m/min; TMH: 17 m/min) beginning at weaning for a period of 8 weeks. The sedentary (SED) rats remained in their cages. The data represent the mean ± SEM (n = 10–12). CAF feeding impaired the shuttle-box acquisition; a p<0.05 vs SED-ST group (LSD after significant repeated measures two-way ANOVA).

Overall as the trials in the shuttle box progressed, the number of avoidances increased, and the escape latencies decreased (trial: F(3,219) = 30.47, p<0.001 and F(3,129) = 60.48 p<0.001, respectively; Fig 3a and 3b). Compared with ST feeding, CAF feeding impaired the two-way avoidance performance; CAF-fed animals showed a smaller increase in the number of avoidances and a smaller decrease in the escape latencies as the trials progressed (Fig 3a; trial x diet interactions: avoidances (F(3,219) = 7.48, p<0.001; escape latencies F(3,219) = 8.59, p<0.01). Paired t-test comparisons indicated that all ST-fed groups and the TMH-CAF group had an increase in the number of avoidances in the last 10-trial block compared with the first block and that all groups except the SED-CAF group had a decrease in the average escape latency of the last block compared with that of the first one (Fig 3).

**Fig 4:**
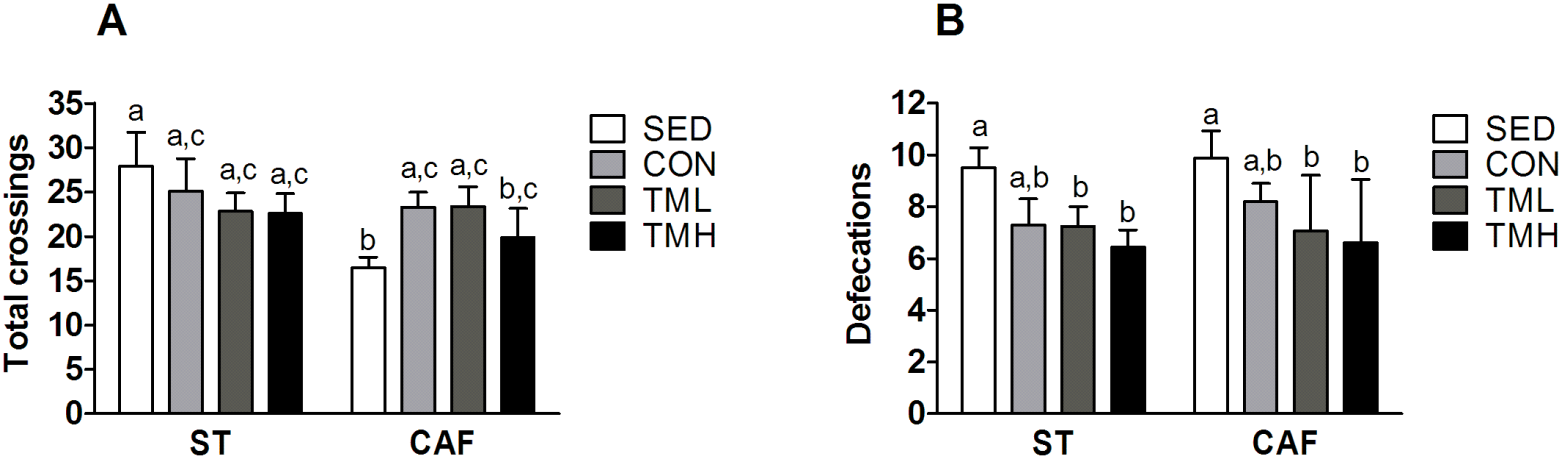
Total crossings (A) and defecations (B) in the shuttle box. Rats were fed standard chow (ST) or a cafeteria diet (CAF) and/or trained on a treadmill (CON: 0 m/min; TML: 12 m/min; TMH: 17 m/min) beginning at weaning for a period of 8 weeks. The sedentary (SED) rats remained in their cages. The data represent the mean ± SEM (n = 10–12). CAF feeding reduced the total crossings, and treadmill intervention reduced the defecations. Treadmill intervention showed a gradual tendency to increase the crossings in the CAF-fed animals (DxT interaction: p = 0.098). ^a, b^Mean values with different letters were significantly different between groups (two-way ANOVA and LSD post hoc comparison, p<0.05).

In addition, CAF feeding caused an overall decrease in the number of crossings in the shuttle box (diet: F(1,80) = 4.175, p<0.05; Fig 4a), and this effect was residually reversed by treadmill intervention (diet x treadmill interaction: F(3,80) = 2.18, p = 0.098; Fig 4a). Treadmill intervention decreased the defecation scores in the shuttle box during two-way active avoidance learning (F(3,81) = 4.79, p<0.01; Fig 4b).

## Discussion

The main observation of the current study was the impairment over time in the shuttle-box session induced by CAF feeding in young adult female rats, suggesting that the CAF diet decreased the capacity to cope with stressful circumstances in which fear and/or threats are induced by a conditioned stimulus [26]. In our experiment, the SED-CAF rats showed fewer avoidance responses and shorter escape latencies than did the SED-ST rats (Fig 3), and that difference was not found in any other CAF-fed group compared with the corresponding ST-fed group (same treadmill intervention). This result demonstrates for the first time that a CAF diet impairs stress-coping strategies and that treadmill intervention partially recovered that deficit. However, that impairment is in apparent discrepancy with some previous studies reporting a reduction in unconditioned anxiety-like behavior in rats fed a palatable CAF diet [12,19] but not with others showing the opposite effect [22,43]. For instance, Alsio et al. [19] reported that the preference for a palatable high-fat diet was inversely associated with high anxiety in tests of exploration of novel environments. Additionally, Warneke et al. [44] reported gender-based differences in the effects, with anxiolysis in CAF-fed female rats and signs of increased anxiety in male rats. Interestingly, the CAF diet was the only diet that reduced the response to chronic variable stress [18] in a study using rats that were fed a high-carbohydrate, high-fat or cafeteria diet for 6 weeks. Together, the observed changes in unconditioned tests of anxiety that resulted from CAF diet consumption are not conclusive and might be caused by differences in experimental factors, such as gender, palatability of the food components, the specific composition of the diet (fat, sugar, and carbohydrates) or even the form of diet presentation (pellet vs lard portions). In contrast, the present experiment demonstrates that CAF feeding induced a passive coping style in the two-way (shuttle-box) active avoidance test to the detriment of an active style response. Moreover, active avoidance behavior is considered a coping defensive behavior that changes dynamically depending on the proximity of the threat and the appraisal analysis. When the threat is inevitable, a conditioned stimulus elicits defensive attentive freezing; however, if the organism realizes there is a possibility to actively avoid the developing threat, startle, freezing or passive avoidance responses can be inhibited in preparation for active avoidance [45,46]. In line with this possibility, the present results suggest that cafeteria diet consumption might modify the appraisal analysis by blocking the preparation of active avoidance. A recent report showed that a cafeteria diet consistently disrupted the trace fear conditioning in adult male rats [47], in agreement with this explanation, although further experiments are needed for confirmation.

However, Ramirez et al. [48] have very recently demonstrated that rats previously trained for two-way active avoidance showed impaired performance after the disconnection of the basal amygdala-nucleus accumbens pathway, revealing that this pathway is involved in stress-coping adaptive behavior [48]. It is known that a CAF diet induces hyperphagia [10], and other recent studies reported that rats exposed to a cafeteria diet developed overconsumption, altered eating patterns and compulsivity toward palatable food consumption [14,49,50]. Those processes trigger addiction-like neuroadaptive responses in brain reward and food hedonic circuits [49], which overlap with homeostatic systems related to energy stores and demands [51–53]. Those findings and the present results suggest that the two-way active avoidance behavior impairment observed in the CAF-fed animals could be mediated by alterations in the amygdala-nucleus accumbens pathway. The reports indicating that dietary obesity and palatable food consumption are linked to depressed dopamine neurotransmission [54] and dopamine D1 receptor gene expression in the nucleus accumbens [55] support that explanation. Moreover, inhibition of the GABAA receptor in the nucleus accumbens shell produced hyperphagia [56], and it is known that there are receptors for metabolic hormones in the amygdala that modulate its functioning [57].

The current results also showed that a CAF diet reduced motor activity, as measured by the distance travelled in an open field and by the number of crossings made during the habituation period and inter-trial intervals of the shuttle-box session. This finding is consistent with other studies reporting reduced activity in obese rats [12,14,58] and humans [59]. Intriguingly, less active animals usually learn two-way avoidance behavior later than do more active animals, indicating that decreased motor activity might also mediate the CAF-induced impairment in this task [60].

Regarding treadmill intervention, the highest significant improvement in two-way active avoidance performance among the CAF-fed groups was found in the TMH-CAF group, indicating that treadmill intervention at higher intensity partially reversed the CAF-induced impairment in that task. The current results also showed that treadmill intervention reduced the defecation during the two-way active avoidance task and increased the distance travelled in the open field. Moreover, the TMH-CAF group that trained at 17 m/min had spent the greatest amount of time in the center of the open field at the end of the 30-min test (Fig 2). Decreased defecation, increased exploration and increased time spent in open spaces have been associated with diminished anxiety-like behavior [61], thus supporting the treadmill-induced decrease in the anxiety levels of the TMH-CAF animals. The literature on rodent studies of the effects of treadmill intervention indicates different outcomes depending on the intensity and/or duration of the exercise. For example, vigorous-intensity exercise (20 m/min, 45 min/day, 5 days/week for 18 weeks) did not change anxiety-like behavior, as measured in the elevated plus-maze, the open field, social interaction and conditioned freezing [62]. By contrast, treadmill exercise protocols of low (12 m/min, 30 min/day) or middle-intensity (14 m/min, 60 min/day) led to a reduction of anxiety-like behavior in the elevated plus-maze and the open field tests [34,35], as well as in the light-dark test [63]; however, the absence of effects have also been reported [37]. Regarding the effects in conditioned tests, we have recently reported that low-intensity treadmill exercise (12 m/min, 30 min/day, 32 weeks) improved two-way active avoidance behavior in both male and female chow-fed adult rats, thus resulting in better conflict coping strategy [36]; this finding is consistent with evidence from human studies indicating that exercise might be an effective intervention for anxiety disorders [64]. As in the present study we only examined female rats, future studies including male rats may help elucidate gender differential effects of exercise on active avoidance behavior in DIO animals [65].

As expected [9,32,66], the biometric and biochemical analyses revealed that CAF feeding that begins at weaning and continues until late adolescence increased the body weight gain, the relative retroperitoneal adipose tissue weight, and the circulating levels of glucose, insulin, triglycerides and leptin. Treadmill intervention prevented the relative increase in RWAT weight and triglyceride levels produced by CAF feeding and also decreased the circulating levels of NEFAs in CAF-fed animals. This result is consistent with the decreased abdominal fat and adipocyte hypertrophy found in DIO rodents after treadmill intervention [32,66] and with the diminished circulating free fatty acid levels found in treadmill-exercised compared with sedentary CAF-fed mice [66]. Furthermore, at higher intensity, the current treadmill intervention partially reversed the increase in serum leptin levels produced by the CAF-diet consumption. Hyperleptinemia is a characteristic manifestation of obesity in humans and rodents that develops with resistance to the action of leptin because the elevated circulating levels of this adipokine can no longer effectively perform its regulatory anorectic function [67–69]. The increased serum leptin levels, RWAT weight (%), body weight gain and carbohydrate and energy intake found in CAF-fed animals compared with their lean counterparts agree with a dysregulation of leptin signaling in the CAF-obese animals. Furthermore, the loss of positive correlations between serum leptin levels and the average daily intake of carbohydrates when only CAF-fed groups were considered in the analysis would reinforce this hypothesis. Interestingly, the decrease in both the circulating levels of leptin and the relative RWAT weight together with the lower energy intake observed in the TMH-CAF animals compared with the non-runner CAF groups would suggest that treadmill exercise at higher intensity might partially prevent leptin resistance. These results are consistent with a net loss of visceral adipose stores and with a 35% reduction in leptin levels previously reported in exercising DIO rats [70]; the findings also agree with the study of Patterson et al. [71] reporting that in inherently leptin-resistant rats that were selectively bred to develop DIO, postweaning exercise caused resistance to obesity on a high-energy diet [71]. Moreover, the threshold for sweet taste has also been related to the action of leptin on taste cells such that an increase in plasma leptin levels activates the leptin receptor, thus reducing the sweet taste information conveyed to the brain. This action of leptin is specific for the inhibition of peripheral gustatory neural and behavioral responses to sweet substances without affecting responses to sour, salty and bitter substances [72,73]. In addition, a diurnal variation in the response to sweet compounds in parallel with that for leptin levels has also been reported in non-obese rodents and humans, but not in obese subjects [72]. Additionally, licking behaviors in response to sucrose and saccharin in rodents were decreased after injection of leptin in obese leptin-deficient and lean normal littermates, but not in obese receptor-deficient mice [74]. In accordance with these findings, it is tempting to speculate that the increase in carbohydrate and sugary milk intake found in CAF-fed rats could be due to an increase in the sweet threshold in our animals (i.e., mainly in the SED-CAF and CON-CAF groups); similarly, a partial prevention of that leptin-mediated effect as a result of treadmill intervention could also contribute to the treadmill-induced decrease in the cumulative sugary milk intake and carbohydrate consumption shown by the TMH-CAF group. Additional experiments aimed at measuring sweet thresholds in ST-fed and CAF-fed animals are needed to further explain that hypothesis; as far as we know, no studies have addressed this issue.

In conclusion, the major finding of the present study is that CAF feeding impairs adaptive coping strategies in young adult female rats as measured by active avoidance behavior. This effect can be partially reversed by treadmill exercise at higher intensity during CAF diet consumption, thus suggesting that exercise might be an effective intervention for anxiety disorders.
